# Case of Femoral Aneurism; Ligature Applied to the External Iliac Artery; Recovery of the Patient

**Published:** 1832-01

**Authors:** Bransby Cooper

**Affiliations:** Surgeon to St. Thomas's Hospital, &c.


THE LONDON

### Medical and Physical Journal.

395, VOL. LXVII.] JANUARY 1832. [67, *New Series.*

"For many fortunate discoveries in medicine, and for the detection of numerous errors, the world is indebted to the rapid circulation of Monthly Journals; and there never existed any work to which the Faculty, in Europe and America, were under deeper obligations than to the 'Medical and Physical Journal of London,' now forming a long but invaluable series."—RUSH.

### ORIGINAL PAPERS, AND CASES,

### OBTAINED FROM PUBLIC INSTITUTIONS AND OTHER AUTHENTIC SOURCES.

### FEMORAL ANEURISM

*Case of Femoral Aneurism; Ligature applied to the External Iliac Artery; Recovery of the Patient.* By BRANSBY COOPER, Esq. F.R.S., Surgeon to ~~St. Thomas's~~ Guy's Hospital, &c.*

THE propriety of tying the external iliac artery in cases of femoral aneurism, is established in proportion to the number of cases in which this mode of treatment is attended with complete and permanent success. I am, therefore, induced to give publicity to the following case, in which relief was obtained under unfavorable constitutional circumstances, as it may tend to give confidence to practitioners in the efficacy of the means of cure adopted.

Hans Jacobs, aged forty-four years, by birth a Dane, has passed the greater part of his life in the laborious occupation of a sailor; has been seven years in the English service, on board a man of war; and, since that, twenty-six years on board different mercantile vessels trading from this country to the East and West Indies. In 1809 he was in the naval hospital at Deal, with dysentery, and in 1825 he was in hospital at Bombay, with a fever; since which his health has been generally good.

It was during his last homeward voyage from Calcutta, in a leaky vessel, that he was called upon to take his turn at the pump every eight hours: when on this duty, on the 2d of April last, without using any extraordinary exertion, he suddenly felt a sensation in his left thigh, (to use his own words,) "as if the limb had been taken clean off by a shot." Immediately he was unable to stand on it, and felt a severe

* We are indebted to our friend, Dr. JAMES JOHNSON, for the transmission of this valuable communication.—EDITOR.

pain in the lower two thirds of the left thigh, extending down the calf of the leg to the ankle. Four or five days after this, a swelling began to make its appearance in the inner and anterior part of the thigh, about two inches below Poupart's ligament: on the appearance of the swelling, the pain began to subside, but was reproduced on his attempting to stand on the limb. At this period he detected a long, narrow pulsating tumor, about the size of his thumb. This tumor continued about two months without any apparent increase; but, on using more exertion than usual, it induced pain and an œdematous state of the leg. During the last three weeks the tumor has rapidly increased in size, accompanied with severe pain, especially about the knee-joint, and for the last week the pain has increased on the outer part of the thigh.

He was admitted into Guy's Hospital 16th July, 1831, having just landed from his ship, the Lord Hungerford, when the following appearances were observed: A large pulsating tumor is situated below Poupart's ligament, towards the inner part of the thigh: it is of an oval figure, the long axis extending in a horizontal direction, being rather broader from side to side than from above to below. The circumference of the tumor is solid; the central part is most prominent, and indicates fluid contents; it is tense, and can be considerably lessened in size by pressure: as it again enlarges, it extends in a direction upwards, so as to overlap Poupart's ligament. The skin is not discoloured over the tumor, but is traversed by large veins, which extend around the limb towards the hip. The whole limb is considerably swollen and enlarged; its temperature increased, the veins congested, and the leg œdematous.

His health is much impaired: he has loss of strength, appetite, and latterly of sleep; pulse sixty-six, natural in its beat; countenance sallow, and expressive of suffering.

July 17th. The following aperient was administered preparatory to an operation: ℞. Pil. Hydr., Ext. Colocynth. comp. āā gr. v. fiant pilulæ duæ, statim sumendæ. Mist. c̄ Magnes. Sulph. bis die.

July 19th, ten A.M. Passed a quiet night; bowels have been freely evacuated; tongue moist and clean; pulse eighty-four, natural; skin cool and moist; no change in the appearance of the aneurism or limb.

*Operation.* The patient was placed on a convenient table in a horizontal position: an incision was made, commencing half an inch above and to the outer side of the external abdominal ring, and extending outwards to within an inch of the anterior superior spinous process of the ileum, in a slightly crescentic direction, the convexity of which was downwards towards Poupart's ligament, and the concavity upwards towards the abdomen. The object to be obtained by this incision was to expose the tendon of the external oblique muscle, but in this case it was so covered with adeps that it required several incisions before it could be completely cleared. This done, by the next incision the tendon of the external oblique was divided precisely in a direction corresponding with the course of the first incision. This semilunar edge of the tendon of the external oblique was now lifted up by an assistant, in order to expose the internal ring; but, from the great developement of the muscular fibres of the internal oblique, where they arise from Poupart's ligament, this object could not be effected, the ring being completely concealed by them: it was, therefore, necessary to pass a director underneath these muscular fibres, and separate them from their origin by means of a blunt-pointed bistoury.

The spermatic cord and internal ring were now exposed. The spermatic cord was then drawn upwards and inwards by an assistant; the fore finger of the right hand was passed into the internal ring, in order to separate the peritoneum from the iliac vessels, by pressing it upwards into the abdomen. The fascia which connects the iliac vein to the artery on the inner side was next separated, in order to make a free passage for the aneurismal needle, which was then passed from within outwards around the artery, armed with a silk ligature, of double the usual size.

The object in using a large ligature was to prevent a too-quick separation before so large an artery is properly secured by the adhesions of its internal coat. From some experiments, I have found that a very fine ligature will ulcerate a large vessel so quickly, that it will sometimes cause, and at all times lead to the danger of after-hemorrhage.

Before the ligature was tightened, the artery was brought to view; a precaution which in this instance was particularly necessary, as a small branch of the external spermatic nerve was seen taking its course along the front of the vessel, and this had to be carefully separated, to prevent its being included in the ligature, which was afterwards tightened. The pulsation in the aneurism immediately ceased, and the size of the tumor lessened at least one third: the edges of the wound were then brought together; one suture was employed to maintain their apposition, and the remainder was secured with adhesive plaster and a dossil of lint. The operation lasted twelve minutes from the first incision to its conclusion.

Half-past two P.M. Complains of a smarting pain in the wound, and is rather restless: pulse seventy-four, and softer; the limb warm; no pulsation in the tumor, which is greatly diminished in size.

Four P.M. Slight pain in the inner part of the thigh; pulse seventy-two, natural; the limb much cooler. Warm flannels and gentle friction to be applied to the limb, and a hot bottle to the foot.

Six P.M. Great pain along the inner part of the thigh; restlessness increased; the temperature of the limb greater. —℞. Tinct. Opii, ♏xxv. statim.

Half-past nine P.M. Pain and restlessness abated; temperature of the limb unaltered; slight numbness of the leg.

July 20th, one A.M. Pain subsided, excepting slight pain in the wound from the motions of the abdominal parietes in respiration, and from pressure: complains of his inability to change his position in bed; sleeps at intervals; the thigh warmer; the leg rather cooler than the opposite limb.

Four A.M. Sleep disturbed, and rests but for short intervals; pulse seventy-eight, soft, and rather fuller; pain in the abdomen from pressure subsided.

Ten A.M. Has slept at intervals since four o'clock; limb warm; tongue clean and moist; pulse one hundred; tumor lessened to one half the size it was previous to the operation. White wine, ℥iv. in the day.

One P.M. Temperature of the skin increased; heat of the limb above its natural standard; tenderness about the wound extending over the iliac towards the lumbar region.

Three P.M. Great restlessness; bowels not evacuated since the operation.—℞. Tinct. Opii, ♏xxv.; Ol. Ricini, ʒiij. statim.

Seven P.M. Sleeps at intervals; pulse ninety-six, not so full; tongue moist; temperature of the limb natural; feels perfectly easy.

Ten P.M. Asleep.

July 21st, three A.M. Complains of heat and tenderness in the lumbar region; bowels not evacuated; skin hot.—℞. Mist. Magn. c̄ Magn. Sulph. ℥j. quâque quartâ horâ donec alvus soluta sit.

Nine A.M. Bowels have been twice evacuated, motions natural; slept at intervals during the night; tongue moist and clean; pulse ninety-six, soft. The œdematous swelling of the leg entirely disappeared; no pulsation to be felt in the tumor; the thigh is reduced to its natural size; slight tenderness in the left iliac region, which is superficial and circumscribed to a small spot. Fotus Papav. c̄ Flor. Anthem. partibus affectis applic.

Six P.M. Slight discharge from the wound, of a serous character; slight pain remains in the neighbourhood of the wound; pulse 102, full and jerking.

July 22d, ten A.M. Has passed a comfortable night; temperature of the limb natural; bowels evacuated during the night; tongue moist; less tenderness in the neighbourhood of the wound; pulse eighty-four, soft and natural, indicating a want of power.

One P.M. Wound dressed; discharge slight, and of a serous character. Wine to be continued, and to have a mutton-chop for dinner. A poultice to be applied to the part feeling tender.

Ten P.M. Free from pain; in other respects as before.

July 23d, noon. Passed a comfortable night; bowels regular; pulse seventy-six, full, but compressible; tongue moist; temperature of the limb natural; free from pain, excepting around the heel and ankle from position.

July 24th, eleven A.M. Has slept well; free from pain, and expresses himself to be in as good health as he ever was in his life. The discharge from the wound healthy, and in greater quantity; tongue moist and clean; pulse seventy-two, soft and natural.

Since the above period the wound continued favorable, and perfectly healed. He has, however, been attacked, in his convalescent state, with a violent inflammation in his right testicle, which only yielded, in the course of a week, to strict antiphlogistic discipline: he then had a similar attack, without any apparent cause, in the left, but which required much less active means to subdue it. He may now be considered in a perfect state of convalescence. The ligature came away the twenty-second day after the operation.

