# Erratum

**Published:** 1832-01

**Authors:** 


ERRATUM.—In the title to the first article, for "St. Thomas's Hospital," read "Guy's."

